# The value of elastography strain rate ratio in benign and malignant BI-RADS-US 3-4 breast masses

**DOI:** 10.17305/bb.2023.9878

**Published:** 2024-06-01

**Authors:** Xinping Li, Weixu Sun, Hongbiao Zhang

**Affiliations:** 1 Department of Ultrasonography, The First People’s Hospital of Yuhang District, Hangzhou, China

**Keywords:** Ultrasound elastography (UE), breast masses, strain rate ratio (SR), Breast Imaging Reporting and Data System (BI-RADS) classification, receiver operating characteristic (ROC) curve

## Abstract

In the realm of breast diagnostics, Breast Imaging Reporting and Data System (BI-RADS) serves as a systematic framework, guiding a methodical exploration into the nuanced narratives of masses. The current study aims to investigate the value of strain rate ratio (SR) of elastography for benign and malignant breast masses categorized by BI-RADS for Ultrasonography (BI-RADS-US) category 3-4. Ultrasonographic data of 1099 breast masses that underwent both elastography and pathological examination were retrospectively analyzed by non-parametric test, consistency analysis, and receiver operating characteristic (ROC) curve sequentially. ROC curve was used to evaluate the diagnostic effect of the SR method in different BI-RADS categories of breast lesions. The maximum Youden index obtained from the ROC curve was 0.845. At a cut-off value of 3.57, the SR values’ diagnostic sensitivity, specificity, accuracy, positive predictive value, and negative predictive value for distinguishing benign and malignant breast lesions were 85.7%, 98.8%, 93.6%, 97.9%, and 91.45%, respectively. Consistency analysis showed that the consistency between SR value and pathological diagnosis was 93.6% (κ ═ 0.864). In addition, the SR values between benign and malignant lesions of BI-RADS 3 and 4 were statistically different (*P* < 0.001). ROC analysis indicated that the diagnostic area under the curve (AUC) for SR value in BI-RADS 3, 4a, 4b, and 4c lesions were 0.985, 0.866, 0.793, and 0.916, respectively. In addition, the study observed 58 cases of missed diagnosis and 20 cases of misdiagnosis in evaluating benign and malignant breast lesions. The elastic SR ratio method has a good diagnostic value for the evaluation of breast masses, particularly for lesions with a score of 3-4. The elastic SR ratio method is instrumental in enhancing the accuracy of diagnosis.

## Introduction

Breast cancer is the most common malignant tumor in women with the highest incidence rate [1]. Nearly one in four women with malignant tumors worldwide has breast cancer [2]. Breast cancer has the highest mortality among female malignant tumors. Currently, there has been a progressive rise in the prevalence and fatality rates of malignant tumors affecting the female breast on a global scale [3]. At present, there is still a lack of primary prevention methods for breast cancer. Early detection, early diagnosis, and early treatment are the keys to the prevention and treatment of breast cancer [4].

Ultrasound diagnosis of breast lesions can be performed according to the size, shape, and boundary of the lesion [5]. In 2003, the American College of Radiology (ACR) proposed a standardized breast imaging reporting and data system for ultrasonography (BI-RADS-US) classification system [6]. However, distinguishing between BI-RADS-US 3 and BI-RADS-US 4 lesions, especially BI-RADS-US 4a, remains a clinical challenge [7]. A prospective study by Barr et al. [8] showed that the malignant rate of BI-RADS 3 lesions was low (0.8%), with only 0.1% showing suspicious malignant changes after six months of follow-up. In another retrospective study, 3% of BI-RADS-US 3 lesions were diagnosed as malignant, resulting in delayed cancer diagnosis in many patients [9]. While a significant number of BI-RADS-US 4a lesions are benign, they still result in excessive biopsies [7]. Therefore, the prediction methods of malignant tumors in BI-RADS-US 3 and 4 lesions should be further explored.

In 1991, American scholar Ophir et al. [10] proposed the concept of elastography, offering a novel approach for differentiating between benign and malignant breast tumors. The basic principle of ultrasound elastography (UE) is to apply stimulation to the tissue. According to the degree of tissue deformation, the echo signals before and after compression are converted into real-time ultrasonic images, and qualitative and quantitative analysis is performed [11]. Strain elastography (SE) is an effective examination method for the diagnosis of liver lesions, thyroid cancer, and breast cancer [12, 13]. In 2013, the revised BI-RADS-US classification system showed that as a feature of malignant masses, lesion hardness information should be included in the diagnosis of breast tumors [14]. Studies suggest that SE, providing additional diagnostic information like colorimetric maps, aids in evaluating of breast BI-RADS-US 3 lesions. It enables examiners to distinguish observable low-risk lesions from high-risk ones requiring immediate biopsy due to increased breast cancer risk [15]. As a semi-parametric quantitative value, the elastic strain rate ratio (SR) can reduce the operator’s subjective bias [16]. In recent years, clinical applications of elastic SR method suggest its high diagnostic value for breast lesions, although its auxiliary value for BI-RADS-US classification system remains a topic of debate [17, 18]. This study intends to use SR as a diagnostic auxiliary information for BI-RADS-US category 3 or 4 lesions, and to explore its auxiliary value in evaluating benign and malignant breast masses.

## Materials and methods

### Analysis of the research objectives

According to inclusion and exclusion criteria, we collected data of 938 patients with a total of 1099 lesions from June 2018 to June 2022 at our hospital. These patients underwent routine ultrasound and UE. The pathological results of the lesions were obtained after biopsy or surgical resection. All patients were female, ranging in age from 14 to 87 years.

Inclusion criteria were as follows: 1) Presence of breast space-occupying lesions identified through two-dimensional ultrasound; 2) SE examination and measurement of SR value; 3) Availability of surgical or biopsy pathological diagnosis results; 4) Provision of signed informed consent.

Exclusion criteria were as follows: 1) Patients with distant metastasis; 2) History of prosthesis implantation; 3) Pregnant or lactating women; 4) Patients with comprised heart and lung function, unsuitable for biopsy; 5) Patients whose SE quality was insufficient for clear analysis.

### Instruments and methods

#### Strain elasticity imaging examination

The Resona R9T ultrasonic diagnostic instrument, equipped with a linear array probe operating at a frequency range of 7.5–13 MHz, was utilized in our procedures. Patients were positioned to fully expose their breasts and bilateral armpits, facilitating standardized scanning and elastography analysis. Upon detection of mass, its two-dimensional image characteristics were observed and evaluated. We then switched to elastography mode, ensuring that the pressure and frequency of applied pressure remained within the normal range during the procedure. When the stable elastic image was obtained, the elastic SR was calculated by the instrument. When selecting and adjusting the region of interest (ROI), it was ensured that the ROI encompassed the entire lesion, while avoiding the cystic area, thick calcified areas of the lesion, and the thick vessels as much as possible. ROI sampling frame is at least twice greater than lesion range. When calculating the elastic SR value, the elastic SR calculated by the ROI where the lesion is located (denoted as A), and the elastic SR calculated by the control ROI (denoted as B) were used. The elastic SR value is determined as B/A, using the same layer of breast gland as a reference [19].

#### Image analysis

Picture archiving and communication systems (PACSs) were used to store image data. After consolidating the two-dimensional ultrasound and elastography images of all lesions, two physicians, each with over two years of experience in elastography examination and more than five years in conventional ultrasound examination, conducted a unified evaluation of the BI-RADS-US categories for these lesions. These physicians obtained a comprehensive joint opinion. Furthermore, both physicians performed a retrospective review of the cases, along with a concordance assessment. The outcomes of this retrospective review were found to be consistent. Based on this, the lesions of BI-RADS categories 2–5 were included in the study. The elastic SR is collected according to the values automatically given by the instrument.

#### Pathological diagnosis

Conventional ultrasound was used to observe the location and size of breast lesions, followed by selection and marking of the appropriate puncture point. After disinfection of the skin at the puncture site, 2% lidocaine was administered locally for anesthesia. The puncture was then performed under color Doppler ultrasound guidance. The puncture tissue length for the 16G Bard’s needle was set to either 15 mm or 22 mm, depending on the lesion’s size and location. A sampling length of 15 mm was used if the lesion’s diameter in the needle direction was less than 15 mm. Conversely, if the diameter of the lesion exceeded 15 mm and the patient’s glandular layer was thicker, a 22 mm sampling length was utilized. Ultrasound-guided puncture was performed to remove the lesion tissue. Each patient was undergoing 2–3 tissue extractions. The extracted tissues were immediately placed in 10% formaldehyde for fixation and further examination. After the puncture procedure, pressure was applied to the puncture point to stop bleeding.

### Ethical statement

Current study was approved by the Ethics Committee of Yuhang First People's Hospital and obtained the ethics committee approval of biomedical research involving humans.

### Statistical analysis

SPSS 22.0 software package (SSPS Inc, Chicago, IL, USA) was used for statistical analysis. Non-normally distributed variables were presented as medians (interquartile range [IQR]). The Mann–Whitney *U* test was used to compare the median of independent sample reduction, and *P* < 0.05 was considered statistically significant. The receiver operating characteristic (ROC) curve was constructed, and the Youden’s index was calculated to obtain the diagnostic boundary point of the SR ratio method. Taking pathological examination as the gold standard, we calculated the diagnostic sensitivity, specificity, positive predictive value, negative predictive value, and accuracy of the SR method in different BI-RADS category lesions.

## Results

### Diagnostic value of elastic strain rate ratio (SR) of lesions in benign and malignant tumors

**Figure 1.**
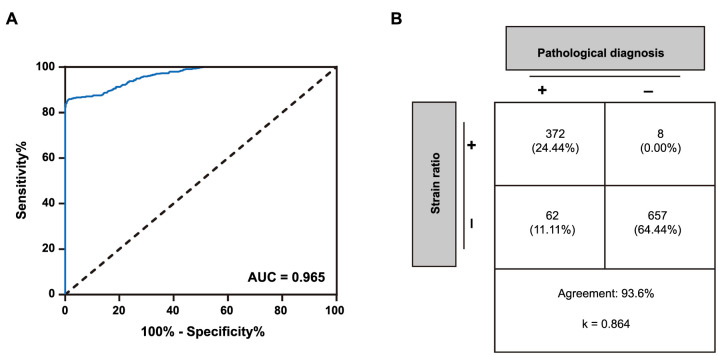
**The diagnostic value of strain rate value of UE in benign and malignant tumors.** (A) ROC curve of strain rate value of UE in the diagnosis of benign and malignant tumors; (B) Consistency analysis of strain rate value of UE and pathological diagnosis results. ROC: Receiver operating characteristic; AUC: Area under the curve; UE: Ultrasound elastography.

Pathological diagnosis of 1099 lesions revealed 434 malignant and 665 benign lesions. The comparative analysis of SR values in benign and malignant tumors is shown in Table 1. The SR value of malignant lesions was significantly higher than that of benign lesions (7.83 [IQR 5.46, 9.69] vs 2.04 [IQR 1.37, 2.69], *P* < 0.001). The diagnostic efficacy of SR value in benign and malignant breast tumors was analyzed by ROC curve. As shown in Figure 1A, the diagnostic area under the curve (AUC) of the SR value was 0.965. The Youden’s index was calculated to be 0.845, and the optimal cut-off value for the SR value was determined to be 3.57. At this diagnostic threshold, the diagnostic sensitivity, specificity, accuracy, positive predictive value, and negative predictive value of SR value in benign and malignant breast tumors were 85.7%, 98.8%, 93.6%, 97.9%, and 91.4%, respectively. When the cut-off value was set at 3.57, the consistency between the SR value and the pathological diagnosis was as high as 93.6% (κ ═ 0.864, Figure 1B).

**Table 1:** Comparative analysis of elastic strain rate ratio in benign and malignant tumors

**Lesion type**	***n***	**Strain rate value (IQR)**
Malignant	434	7.83 (5.46, 9.69)
Benign	665	2.04 (1.37, 2.69)
*Z* value		−26.10
*P* value		<0.001

IQR: Interquartile range.

### Comparative analysis of elastic SR in different BI-RADS classification lesions

The SR values of different BI-RADS classification lesions are shown in Table 2. There were 46 cases of BI-RADS 2 lesions. According to the pathological results, all of the 46 lesions were benign. In addition, the SR ratio of the two categories of lesions was 1.36 (IQR 0.76, 1.80), and the SR ratio of all the two categories of lesions was less than 3.57. In addition, pathological results showed that 306 (98.7%) benign and 4 (1.3%) malignant lesions were included in 310 cases of BI-RADS 3 lesions. Compared with benign lesions, the SR values of malignant ones were significantly higher (*P* < 0.001). In the group of 604 BI-RADS 4 lesions, 312 (51.7%) were benign and 292 (48.3%) were malignant. The SR values of malignant lesions were significantly higher than those of benign lesions (All *P* < 0.001). In addition, all 138 cases of BI-RADS 5 lesions were malignant, with an SR value of 10.01 (8.11, 12.50).

**Table 2:** Elastic strain ratio analysis of different BI-RADS category lesions

**BI-RADS category**	**Lesion type**	***n***	**Strain rate value (IQR)**	***Z***	***P***
Grade 2	Malignant	0	/		/
	Benign	47	1.36 (0.76, 1.80)		
Grade 3	Malignant	4	3.84 (3.15, 4.34)	−3.332	<0.001
	Benign	306	1.66 (0.99, 2.23)		
Grade 4	Malignant	292	6.96 (4.82, 8.63)	−18.417	<0.001
	Benign	312	2.46 (1.90, 3.02)		
Grade 5	Malignant	138	10.01 (8.11, 12.50)		/
	Benign	0	/		

BI-RADS: Breast Imaging Reporting and Data System.

### The auxiliary diagnostic value of elastic SR ratio in BI-RADS 3 and 4 lesions

**Figure 2.**
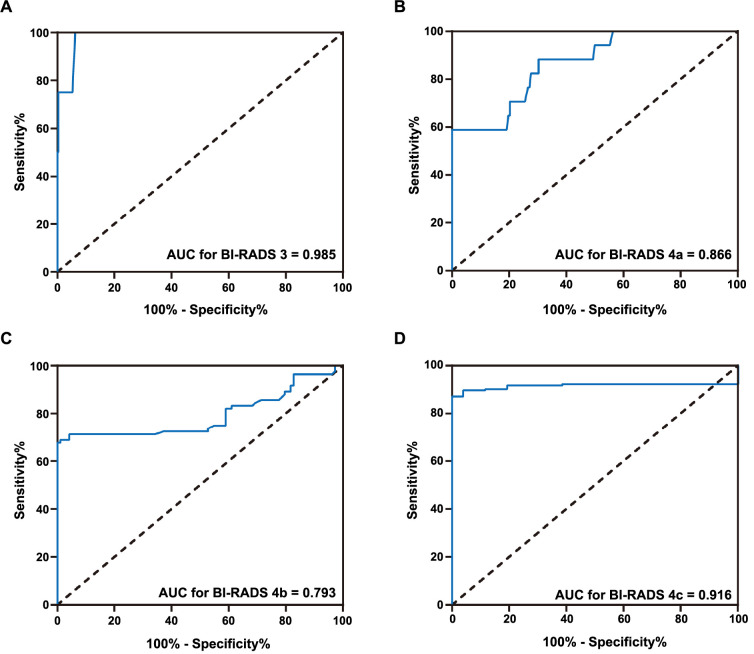
**ROC curve of elastic strain rate ratio in the diagnosis of BI-RADS 3 and 4 lesions**. ROC: Receiver operating characteristic; BI-RADS: Breast Imaging Reporting and Data System; AUC: Area under the curve.

The diagnostic efficacy of SR values in BI-RADS 3 and 4 lesions was analyzed by ROC curves. Among four malignant lesions in BI-RADS category 3, there were three cases of invasive ductal carcinoma (SR ratios were 3.74, 3.93, and 4.47, respectively) and one case of ductal carcinoma in situ (SR ratio was 2.95). As shown in Table 3 and Figure 2, the AUC of the SR ratio method for the diagnosis of BI-RADS 3 lesions was 0.985. With a cut-off value of 2.93, the diagnostic sensitivity of SR value in BI-RADS 3 lesions was 100%, the specificity was 93.8%, and the diagnostic accuracy was 93.9%. In BI-RADS category 4 lesions, ROC curve analysis revealed that the diagnostic AUC of SR value in BI-RADS category 4a, 4b, and 4c was 0.866, 0.793, and 0.916, respectively. Interestingly, the diagnostic specificity of SR values in BI-RADS category 4 lesions exceeded 99%, suggesting a low misdiagnosis rate.

**Table 3:** Diagnostic efficacy of elastic strain rate ratio in BI-RADS category 3 and 4 lesions

**BI-RADS category**	**Cut-off**	**Sensitivity**	**Specificity**	**Positive predictive value**	**Negative predictive value**	**Accuracy**	**AUC**
Grade 3	2.93	100.0%	93.8%	17.4%	100.0%	93.9%	0.985
Grade 4a	3.85	58.8%	100.0%	100.0%	96.4%	96.6%	0.866
Grade 4b	3.57	68.7%	99.0%	98.2%	78.7%	84.9%	0.793
Grade 4c	4.43	87.0%	100.0%	100.0%	51.0%	88.6%	0.916

BI-RADS: Breast Imaging Reporting and Data System; AUC: Area under the curve.

**Figure 3.**
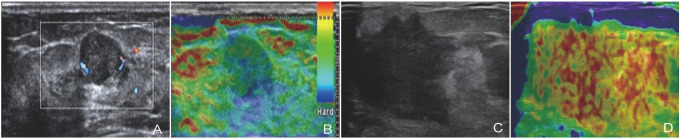
**Conventional ultrasound and UE representative images of breast lesions**. (A and B) Conventional ultrasound and UE images of benign breast lesions; (C and D) Conventional ultrasound and UE images of malignant breast lesions. UE: Ultrasound elastography.

UE and conventional ultrasound images of benign and malignant breast lesions are shown in Figure 3. Among them, the conventional ultrasound images of benign lesions were elliptical with clear borders and homogeneous internal echoes (Figure 3A). The SR value for the UE image was 1.32 (Figure 3B). In addition, conventional ultrasound images of malignant breast lesions displayed irregular morphology, blurred margins, and uneven internal echoes (Figure 3C), with a UE image SR value of 4.47 (Figure 3D).

Based on the cut-off values for different BI-RADS classifications, a total of 58 lesions were missed, including 7 cases of BI-RADS 4a, 26 cases of BI-RADS 4b, and 25 cases of BI-RADS 4c Table 4. Among these missed lesions, invasive ductal carcinoma was the most prevalent accounting for 43.1% of cases. In addition, there were 20 cases of misdiagnosed lesions, including 19 cases of BI-RADS 3 and 1 case of BI-RADS 4b.

## Discussion

Breast cancer is one of the most common malignant tumors in women [1]. Early detection and diagnosis of breast lesions can help to improve the survival rate and quality of life of patients and reduce psychological burden [1, 2]. Early-stage breast cancer is often discovered because patients inadvertently seek medical attention, presenting early as painless, single small breast masses [20]. As the disease progresses, the mass’s surface may become irregular, its boundary with surrounding tissue is less clear, its texture becomes firmer, and the glandular activity is poor [20]. With the increasing incidence of breast tumors, imaging examinations have become essential in diagnosing breast diseases [21]. Currently, commonly used imaging methods in clinical practice are ultrasound, molybdenum target, and MRI. [22]. MRI offers a high diagnostic value for breast diseases and can evaluate the morphology and function of breast lesions. However, its disadvantages include high cost and lengthy procedure times, making it less suitable for routine screening and diagnosis of breast diseases [23]. Molybdenum target X-ray is mainly used for breast cancer screening. Its principle is to make a diagnosis by comparing the density difference between normal breast tissue and lesions. However, its effectiveness is limited for breast lesions in dense breast tissues [23]. Similarly, molybdenum target X-ray is more sensitive to microcalcifications within lesions, but a drawback is its radiation exposure [24]. With the ongoing advancements of ultrasound technology and continuous improvement of instruments, ultrasound has become a vital examination method for breast diseases [25]. Its advantages are clear: it is real time, fast, non-invasive, and cost-effective [25]. Especially in dense breast lesions, ultrasound is an indispensable supplementary means of molybdenum target X-ray [26]. In addition, ultrasound also plays an irreplaceable unique advantage in the identification of cystic and solid breast lesions [27]. Moreover, ultrasound is non-radioactive and is the preferred examination method for pregnant, lactating, and adolescent women [25].

Two-dimensional ultrasound can diagnose and differentiate tumors based on boundary, morphology, posterior echo attenuation, calcification, peripheral and internal blood flow, and resistance index. However, it lacks capability to gather information on tissue stiffness [25]. UE is a novel ultrasound diagnostic technique that can be used to study tumors and diffuse diseases that are undetectable by conventional ultrasound [11]. It can reflect and quantify the elasticity (i.e., hardness) of the tissue [11]. The theoretical basis of elastography in clinical application is based on the physiological and pathological state of tissues. The elasticity (hardness) of biological tissues is closely related to the physiological and pathological changes of organisms [13]. Significant elasticity changes occur in tissues harboring hard masses or tumors. Structures with high elastic coefficient produce relatively small strain, indicating greater hardness. Conversely, structures with low elastic coefficient result in larger strain and comparatively lesser hardness. In recent years, elastography has matured as an ultrasound technique and is widely used in clinical practice. Elastic SR ratio, a semi-parametric quantification method, has been introduced for diagnosing breast masses, but its diagnostic value a subject of debate [28]. Therefore, this study focuses on analyzing the SR values of benign and malignant breast lesions across different BI-RADS classifications and exploring the auxiliary diagnostic value of SR values in clinical breast cancer diagnosis.

The hardness of various breast tissues ranges from low to high, encompassing adipose tissue, normal glandular tissue, fibrotic lesions of the breast, non-invasive ductal carcinoma, and invasive ductal carcinoma of the breast [29]. Malignant breast lesions are typically 2–3 times harder than benign ones [30]. Breast malignant tumors often exhibit infiltrative growth, leading to adhesion to the surrounding glandular stroma. This results in limited movement of lesion tissue and surrounding areas, increased hardness, and higher elasticity coefficient [30]. The elasticity coefficient is closely related to the biological properties of the lesion. Different internal molecular structures of tissues yield varying elasticities. UE can discern characteristics of lesion tissues based on the differences in hardness and the hardness measurement of the lesion can be repeated [30]. A total of 434 malignant lesions and 665 benign lesions were included in this study. Among them, the SR value of malignant lesions was significantly higher than that of benign lesions (7.83 [IQR 5.46, 9.69] vs 2.04 [IQR 1.37, 2.69], *P* < 0.001), aligning with previous studies. In addition, ROC curve analysis showed that the diagnostic AUC of SR value in distinguishing benign and malignant breast lesions was as high as 0.965, suggesting high diagnostic efficiency. With a cut-off value of 3.57, the diagnostic sensitivity of SR value in benign and malignant breast lesions was 85.7%, the specificity was 98.8% and the consistency with pathological diagnosis was as high as 93.6% (κ ═ 0.864). These results suggest that SR value has potential as a diagnostic method for benign and malignant breast lesions.

**Table 4:** The pathological types and proportion of missed diagnosis and misdiagnosis of breast solid masses by strain rate ratio method

**Missed diagnosis (false negative)**		**Misdiagnosis (false positive)**	
**Pathological type**	***n* (%)**	**Pathological type**	***n* (%)**
Invasive ductal carcinoma	25 (43.1)	Intraductal papilloma	9 (42.0)
Ductal carcinoma in situ	16 (27.6)	Fibroadenoma	5 (25.0)
Papillary carcinoma	7 (12.1)	Chronic mastitis	2 (10.0)
Mucinous carcinoma	5 (8.6)	Fibrocystic disease	2 (10.0)
Non-Hodgkin’ s lymphoma	3 (5.2)	Borderline phyllodes tumor	1 (5.0)
Medullary carcinoma	2 (3.4)	Tubular adenoma	1 (5.0)

The American College of Radiology developed a breast imaging reporting and data system in 1992 and established a breast ultrasound diagnostic criterion (BI-RADS-US) in 2003 [6]. The evaluation content of the BI-RADS classification system is complex and often relies on the subjective understanding of the reader. To address this, Zhi et al. [31] proposed a method combining BI-RADS with UE. Based on the BI-RADS classification system, the image of UE was comprehensively considered to discern benign and malignant tumors. They found that when using the SR ratio method to determine the nature of breast masses, the best cut-off point was found at an SR value of 3.05 according to the ROC curve. The corresponding sensitivity, specificity, and accuracy were 92.4%, 91.1%, and 91.4%, respectively [31]. In addition, Farrokh et al. observed that the SR value of benign breast lesions was significantly lower than that of malignant lesions. The sensitivity and specificity of benign and malignant breast lesions were 92.6% and 95.2%, respectively, indicating a diagnostic efficiency superior to conventional ultrasound [32]. In addition, another study by Zhi et al. [33] showed that the specificity and accuracy of combining BI-RADS with UE were higher than using the BI-RADS classification alone, while the sensitivity of the two methods was similar. In addition, the positive predictive value of BI-RADS category 4 lesions significantly improved after combining the BI-RADS classification system with UE [33]. Unlike the elastic scoring method in this study, the SR ratio method was used as the diagnostic criteria for UE. BI-RADS category 2 or 5 breast lesions are benign or malignant, respectively, the auxiliary diagnostic significance of the SR method is not pronounced. However, in BI-RADS 3 and 4 lesions, the SR values of malignant tumors were higher than those of benign tumors, with a statistically significant difference (all *P* < 0.001).

In clinical practice, the clinical pathways of category 3 and 4 lesions differ significantly. In this study, the diagnostic efficacy of SR value in BI-RADS 3 and 4 lesions was analyzed by ROC curve. With a cut-off value of 2.93, the diagnostic sensitivity of SR value in the three lesion categories was 100%, indicating no missed diagnosis. In addition, the diagnostic specificity and accuracy were 93.8% and 93.9%, respectively. For 4a, 4b, or 4c lesions with SR values exceeding 3.85, 3.57, or 4.43, respectively, the diagnostic specificity was over 99%, suggesting a low risk of misdiagnosis. Biopsying only lesions below the cut-off value could significantly reduce the false-negative rate and avoid unnecessary biopsies. However, for 4a and 4b lesions, the diagnostic sensitivity of SR value was only 58.8% and 68.7%, indicating a higher risk of missed diagnosis. Therefore, 4a and 4b lesions below the cut-off value still need further pathological examination for definitive diagnosis.

Pathological factors of the lesions impact the diagnostic accuracy of UE, including the proportion of cellular and fibrous components, calcification, myxoid degeneration, hemorrhage and necrosis, etc. The elastic coefficients of different tissues overlap to some extent, which can lead to diagnostic errors when using UE alone for differentiating benign from malignant breast tumors. In this study, 78 lesions were inconsistent with pathological findings, including 58 missed lesions and 20 misdiagnosed lesions. Among the missed lesions, invasive ductal carcinoma was the most common (43.1%). This type of carcinoma, also known as colloid carcinoma, is often characterized by abundant interstitial and cytoplasmic mucin with relatively weak invasion and softer texture, which can lead to misdiagnosis. In addition, the malignant lesions of invasive ductal carcinoma are easy to infiltrate and grow into the surrounding tissues, resulting in a corresponding increase in the hardness of the surrounding tissues. When measuring the SR value of normal gland tissue at the same depth as the malignant lesion, it is easy to mis-detect the area where the malignant lesion infiltrates into the surrounding tissue. This can result in an elastic strain value inconsistent with the actual hardness of the lesion, leading to a lower final SR value. In addition, 16 cases of ductal carcinoma in situ (27.6%) were missed. Ductal carcinoma in situ typically forms along the mammary duct separated by normal glandular tissue in the middle. Most intraductal lesions are surrounded by local intraductal effusion. These factors are the main cause of missed diagnosis of ductal carcinoma in situ. There were 20 cases misdiagnosed as intraductal papilloma (9 cases, 42.0%). Intraductal papilloma of intraductal carcinoma in situ is rich in fibrous stromal components, which leads to increased hardness of lesions and leads to misdiagnosis.

## Conclusion

In summary, the elastic SR ratio method has a good diagnostic value in evaluating breast masses. Especially for BI-RADS 3 and 4 lesions, the diagnostic accuracy of the SR method is high, which can effectively reduce the misdiagnosis rate and missed diagnosis rate of breast lesions. In addition, the SR method has the potential to become an auxiliary diagnostic method for pathological examination, reducing unnecessary biopsies. This is significantly beneficial for the early diagnosis and treatment of lesions. In addition, studies focusing on the diagnostic ability of other elastography techniques, such as shear wave elastography in BI-RADS-US classification would be of great benefit [34, 35].

## Data Availability

The datasets used and/or analyzed during the current study are available from the corresponding author on reasonable request.
